# Enhanced Handover Decision Algorithm in Heterogeneous Wireless Network

**DOI:** 10.3390/s17071626

**Published:** 2017-07-14

**Authors:** Radhwan Mohamed Abdullah, Zuriati Ahmad Zukarnain

**Affiliations:** Department of Wireless and Communication Technology, Faculty of Computer Science and Information Technolog, University Putra Malaysia, Serdang 43400, Malaysia

**Keywords:** vertical handover, decision algorithm, handover operation, mobility management, performance analysis

## Abstract

Transferring a huge amount of data between different network locations over the network links depends on the network’s traffic capacity and data rate. Traditionally, a mobile device may be moved to achieve the operations of vertical handover, considering only one criterion, that is the Received Signal Strength (RSS). The use of a single criterion may cause service interruption, an unbalanced network load and an inefficient vertical handover. In this paper, we propose an enhanced vertical handover decision algorithm based on multiple criteria in the heterogeneous wireless network. The algorithm consists of three technology interfaces: Long-Term Evolution (LTE), Worldwide interoperability for Microwave Access (WiMAX) and Wireless Local Area Network (WLAN). It also employs three types of vertical handover decision algorithms: equal priority, mobile priority and network priority. The simulation results illustrate that the three types of decision algorithms outperform the traditional network decision algorithm in terms of handover number probability and the handover failure probability. In addition, it is noticed that the network priority handover decision algorithm produces better results compared to the equal priority and the mobile priority handover decision algorithm. Finally, the simulation results are validated by the analytical model.

## 1. Introduction

The demand of data transfer rate and traffic capacity of mobile communication is growing rapidly; thus, the concept of the heterogeneous network is introduced to meet this demand. In a heterogeneous network, the mobility feature is essential because mobile devices must be able to roam throughout the network and to connect to various radio access technologies. Conventionally, the mobile device is considered the point of attachment based on single criteria, such as Received Signal Strength (RSS). General opinion has been that the simplest algorithm to determine handover is based on RSS [1], but RSS fluctuates, making it unreliable [2]. This is caused by each element in a heterogeneous network having different thresholds of RSS, causing high packet delay, excessive handover, high handover failure probability and decreasing overall throughput in the RSS-based algorithm. Furthermore, vertical handover has several issues that are worth noting. They are as follows:

1. The algorithm should be reliable; an inaccurate vertical handover decision may cost network resources.
2. The algorithm should be able to distribute mobile devices fairly; this is to balance traffic loads on the networks.
3. The algorithm should be accurate; it must be able to identify the need to increase data transfer rate to mobile devices on the network.

Moving on, implementing multiple criteria on the algorithm of vertical handover helps to provide an alternative network that might be the best network target. The rest of this paper is organized as follows: a brief related work of the approaches in the network selection area is given in Section 2, followed by the description of the multiple criteria handover decision algorithm in Section 3. The simulation methodology is described in Section 4. Section 5 presents the results with the related discussions. Section 6 presents an analytical model for validating the simulation results; finally, the conclusions constitute the last section.

## 2. Related Work

There are several methods of vertical handover decision algorithm, as follows:

RSS-based algorithms: This method uses the RSS algorithm as the handover trigger [3] and to decide handover [4]. The RSS-based algorithm has been optimized by adapting the RSS threshold [5] and by combining the RSS threshold with the user’s velocity and location [6].

Context-aware-based algorithms: Handover is decided based on signal quality, the network and the context of the mobile device [1]. Context can be defined as the situation of an entity [7] or a location, environment, identity and time [8].

Cost function-based algorithms: This method can be approached in two ways: network-related cost function and user-related cost function [9,10]. Variables involved in the user-related cost function are security, monetary cost and power consumption [11,12].

Fuzzy logic-based algorithms: The two steps involved are: (a) fuzzification and weighting procedure [13]; (b) decision making. This step uses Multi-Attribute Decision Making (MADM) [14,15].

Multiple criteria-based algorithms: This method combines the multiple criteria-based algorithms to reduce power consumption [16,17,18].

Generally, RSS-based algorithms are the least complex system (Table 1), but it is also the least accurate. Meanwhile, algorithms, such as fuzzy logic and cost function, are highly complex, but they are also highly accurate and provide higher network efficiency. So far, there has been much research done on multiple criteria vertical handover decision algorithms. It is found that they can make a quantitatively-calculated decision using some criteria of the candidates [19]. This conclusion is derived by comparing Multiple criteria Exponent Weighting (MEW), Simple Additive Weighting (SAW), the Technique for Order Preference by Similarity to Ideal Solution (TOPSIS) and Grey Relational Analysis (GRA) [20]. Their performance in handover efficiency is also examined [21,22].

The comparison has been simulated in the heterogeneous network environment of WLAN, UMTS (Universal Mobile Telecommunications Systems) and GPRS. Then, a comparison of network performance (BER, delay, jitter and bandwidth) has been carried out. There is also a comparison and a performance evaluation of SAW and the Weighted Product Model (WPM) in terms of processing delay [23] in the environment of WLAN and Worldwide interoperability for Microwave Access (WiMAX). The results indicate that WPM has better accuracy in choosing a target network compared to SAW. Another multiple criteria algorithm, ELECTRE, has been implemented as the vertical handover decision and evaluated using numerical analysis. ELECTRE (Elimination et Croix Traduisant la Realite, or elimination and choice expressing reality) is compared to the algorithm of SAW and TOPSIS [24,25]. It should be noted that the appropriate choice of the criteria is crucial to ensure decision accuracy. There are many criteria, user-related or network related, such as RSS, mobility, application and bandwidth.

## 3. Multiple Criteria Handoff Decision Algorithm

Moving on, TOPSIS has several advantages over other multiple criteria algorithms. Its concept is simple: it has an efficient computing characteristic and is able to measure relative performance for each alternative [26]. Furthermore, it only requires one subjective input to calculate the decision. During simulation, compared to other algorithms, TOPSIS provides higher throughput and lower packet loss [27]. In a different perspective, the handover decision algorithm is composed of four criteria: RSS, cost function, mobile speed and network occupancy. Moreover, the algorithm needs the network (network topology and radio) and mobile (cost function and mobile speed) as the input. More details about the network parameters are explained in the next section.

There are two mobile station parameters in this study: cost function and mobile speed.

There are three types of cost function, listed as follows:

1. Gold cost: A premier user subscription that allows the use of the highest-level of Quality of Service (QoS). The cost function is irrelevant.
2. Silver cost: A medium priority user subscription that would try to balance between QoS requirements and cost function.
3. Bronze cost: A lower user subscription where the cost function is significantly more important than any QoS parameters.

**Figure 1:**
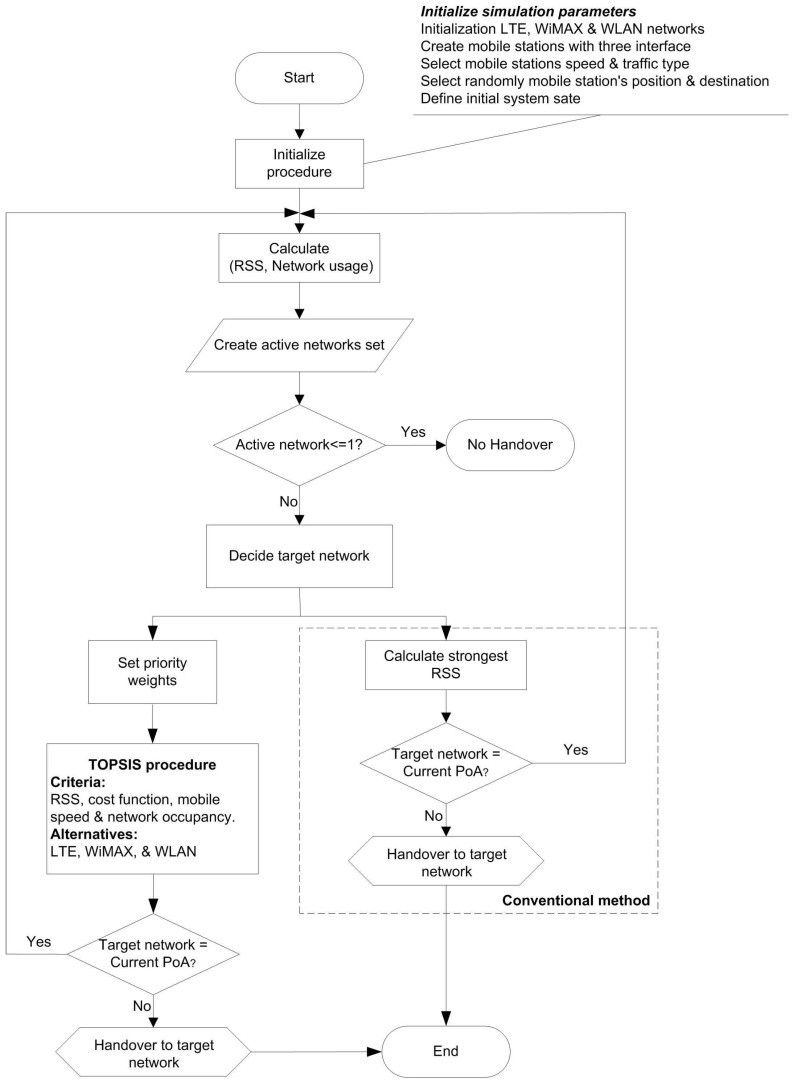
Message flow diagram for the multiple criteria handoff decision algorithm.

There are five different mobile speeds between the low speed vehicular mobile station (5 m/s) and the high speed vehicular mobile station (25 m/s) [25]. Figure 1 illustrates the flowchart of the handover decision algorithm to select the networks.

The TOPSIS method provides flexibility in defining the weights of the multiple criteria priority. There are three types of priority in the multiple criteria vertical handover decision: equal priority, mobile priority and network priority. The priority method emphasizes the mobile parameter (cost function and mobile speed); meanwhile, network priority emphasizes network occupancy. Each priority has a certain weight, as presented in Table 2.

In these three types of priorities, we utilize the weights to compute the expected impact of each criterion on selecting the most appropriate network for handover purposes. This is performed by employing the TOPSIS method to compare between the available candidate networks. Suppose there are M alternatives (options) of candidate networks available for handover selection. Building on the previous discussion, TOPSIS will be the method for selecting the most appropriate network for handover. The steps are as follows [25]:

1. Construct the normalized decision matrix: to transform dimensional attributes into non-dimensional ones. This will allow comparison across criteria, using the following equation:
  (1) $$r_{ij}=\frac{N_{ij}}{\sqrt{\sum_{i=1}^{m}N_{ij}^{2}}}\;\;\;\;\;i=1\ldots m,\;j=1\ldots n$$
  where
  $r_{ij}$ represents the non-dimensional matrix.
    $N_{ij}$ represents the score of option *i* with respect to criterion *j*.
  *m* represents the alternatives (LTE, WiMAX and WLAN).
  *n* represents the criteria (RSS, cost function, mobile speed, and network occupancy).
2. Construct the weights decision matrix. The TOPSIS method is used to multiply each column of the normalized decision matrix by the weights. An element of the new matrix is:
  (2) $$V_{ij}={Wr}_{i}*r_{ij}$$
  where *W* represents the weight
3. Determine the ideal and negative ideal solutions.
  (a) Ideal solution:
    (3) $$A^{+}=\left\{V_{1}^{+},\ldots ,V_{n}^{+}\right\},\;$$
    where $V_{j}^{+}=\left\{\max\left(V_{ij}\right)\;if\;j\in J;\min\left(V_{ij}\right)\;if\;j\in J^{-}\right\}$
  (b) Negative ideal solution:
    (4) $$A^{-}=\left\{V_{1}^{-},\;\ldots ,V_{n}^{-}\right\},$$
    where $V^{-}=\left\{\min\left(V_{ij}\right)\;if\;j\in J;\max\left(V_{ij}\right)\;if\;j\in J^{-}\right\}$
    *J* is the set of benefit attributes or criteria (more is better)
    $J^{-}$ is the set of negative attributes or criteria (less is better)
4. Calculate the separation measures for each alternative.
  (a) The separation from the positive ideal alternative is:
    (5) $$S_{i}^{+}=\sqrt{\sum_{j=1}^{n}\left(V_{ij}-V_{j}^{+}\right)}\;i=1,\ldots .,m$$
  (b) The separation from the negative ideal alternative is:
    (6) $$S_{i}^{-}=\sqrt{\sum_{j=1}^{n}\left(V_{ij}-V_{j}^{-}\right)}\;i=1,\ldots .,m$$
5. Calculate the relative closest to the ideal solution.
  (7) $$C_{i}^{+}=\frac{S_{i}^{-}}{S_{i}^{+}+S_{i}^{-}}\;$$
  where $0<C_{i}^{+}<1,\;\;\;\;i=1,\ldots .,m$
  $$C_{i}^{+}=1,\;if\;A_{i}=A^{+}$$
  $$C_{i}^{+}=0,\;if\;A_{i}=A^{-}$$
6. Rank the preference order.

A set of alternatives can now be preference ranked according to the descending order of $C_{i}^{+}$.

## 4. Simulation Algorithm

To obtain performance results, the proposed algorithm is implemented by using NS-2 simulation (UPM Lib., Selangor , Malaysia) [28]. We implemented the traditional network according to the RSS method in vertical mode. Actually, almost all of the research papers and real deployments are focused on a single metric like RSS because this information is measured in almost all mobile devices when this method is applied for implementation in the handover aspect due to the cost and simplicity. Therefore, to investigate the effectiveness of our proposal, the simulations are conducted in comparison with this product and taken as the benchmark for the analysis of the handover process. After that, an evaluation of the handover efficiency is done by examining the probability of the success and failure of handover.

Moving on, handovers are calculated based on the total vertical handover occurrence during an active call. It is crucial in assessing a mobile network’s performance as it is influenced by signaling load and delivery of QoS. It is worth noting that unnecessary handover will waste network resources and time, consequently contributing to inefficiency. The handover failure probability is the average of the incoming handover requests that cannot be serviced due to the lack of resources.

The network topology consists of the LET, WiMAX and WLAN networks. The radius of WiMAX is 2500 m, LTE 1000 m and WLAN 300 m. WiMAX covers 75% of the simulation area; meanwhile, LTE covers 65%, and WLAN covers 75%. The radio parameter is presented in Table 2. The tracks of MNs are randomly paths [28]. The User Datagram Protocol (UDP) is used to transmit 4960 bytes of video and 320 bytes of audio traffic between MN and CN. Meanwhile, the inter-packet transfer duration is 0.004 s. The simulation time is set at 480 s, while the results are computed by taking the average speed of 10-times executing the scenario. Simulations are done using 15 random mobile node trajectories across LET, WiMAX and WLAN networks.

## 5. Results and Analyses

**Figure 2:**
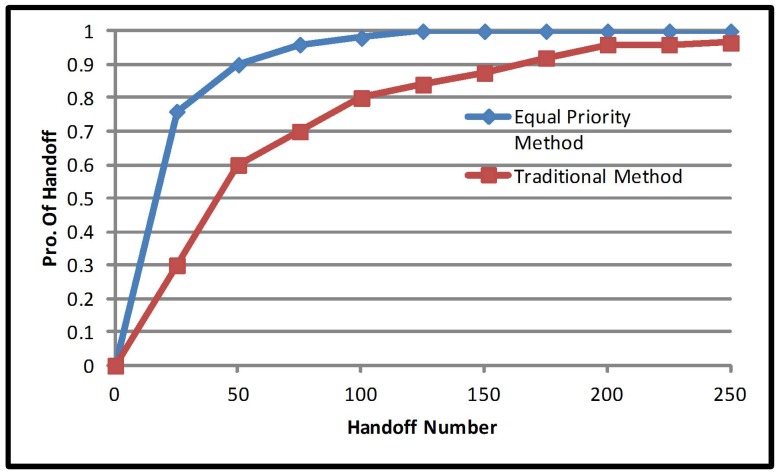
Handoff number probability of the equal priority method.

**Figure 3:**
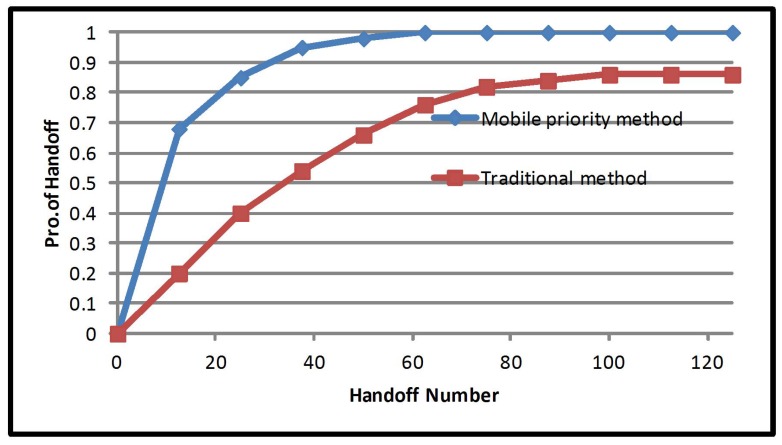
Handoff number probability of the mobile priority method.

Looking at a different point of discussion, there are three types of priorities to be considered in the multiple criteria handover decision. They are equal priority, mobile priority and network priority, and they are implemented in a heterogeneous network environment. Each priority performance is compared to the traditional method, which only considers RSS as its criteria: the multiple criteria method considers the cost function, mobile speed and network occupancy. Figure 2 presents the number of handover allocations of equal priority multiple criteria, for 100 mobile users. Equal priority has been proven to reduce the number of handover by 22.9%; the number of handovers are reduced to 33 from 44 after implementation of equal property multiple criteria. This improves network efficiency and increases resource availability. Figure 3 presents the number of handovers after the implementation of mobile priority multiple criteria. Mobile priority reduced the number of handovers by 40.29%; the number of handovers from 44 to 30 after the implementation of mobile priority multiple criteria.

**Figure 4:**
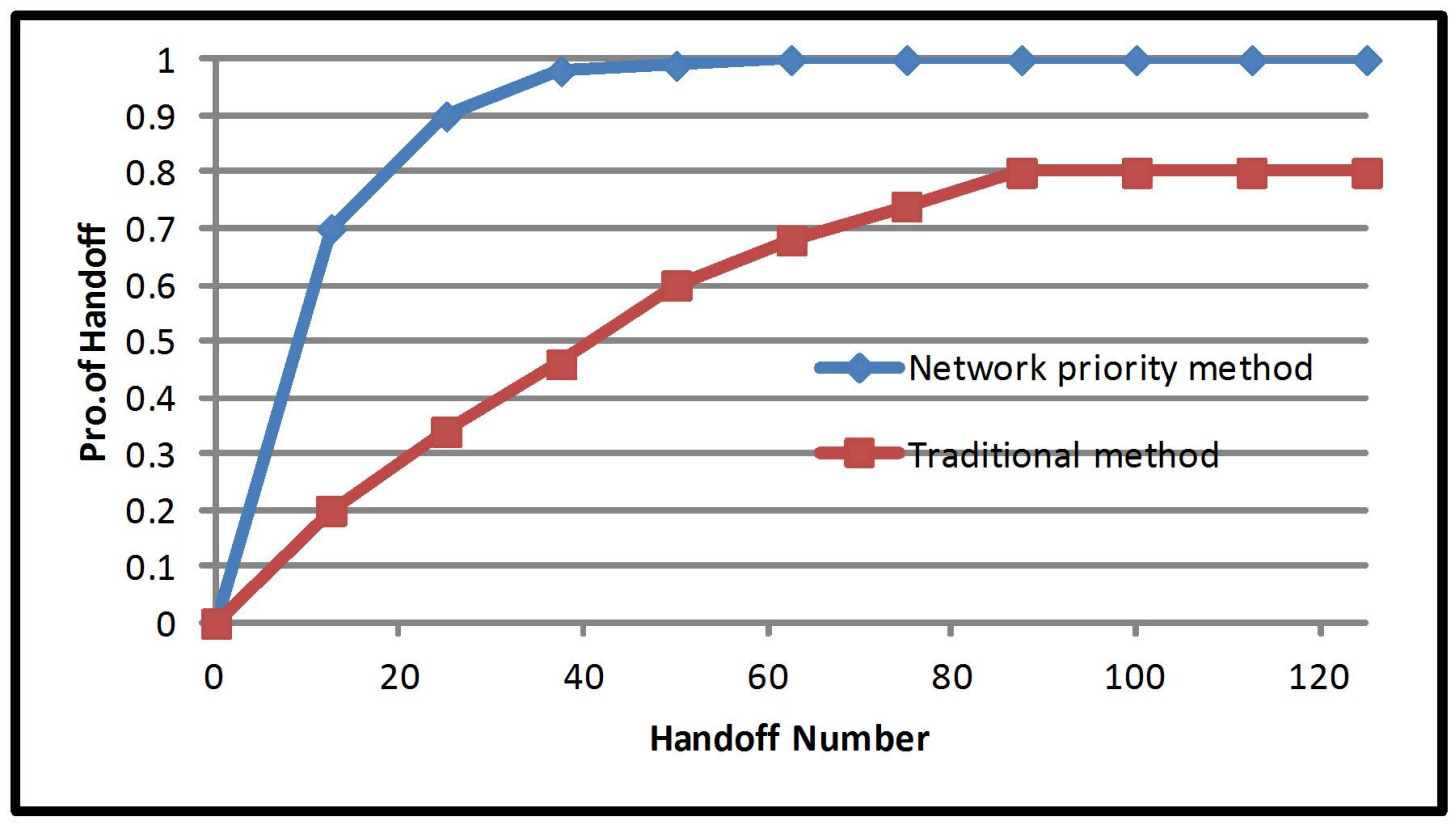
Handoff number probability of the network priority method.

The number of handovers for network priority multiple criteria is presented in Figure 4. The improvement is 60%, where the number of handover averages for traditional method are 44 and the number of handover averages for network priority multiple criteria is 25. Building on the previous discussion, the traditional method of vertical handover decision produces a higher volume of handovers. This leads to the increase of signaling load on the network. As shown in the previous paragraph, the mobile priority multiple criteria method performed better than the equal priority and network priority. The weights of mobile priority multiple criteria have a large ratio on the cost function and mobile speed. Mobile speed is linked to the number of handovers, where mobile users with higher speed are more likely to experience a ping pong effect. This proves that focusing on the cost function and mobile speed reduces unnecessary handovers.

**Figure 5:**
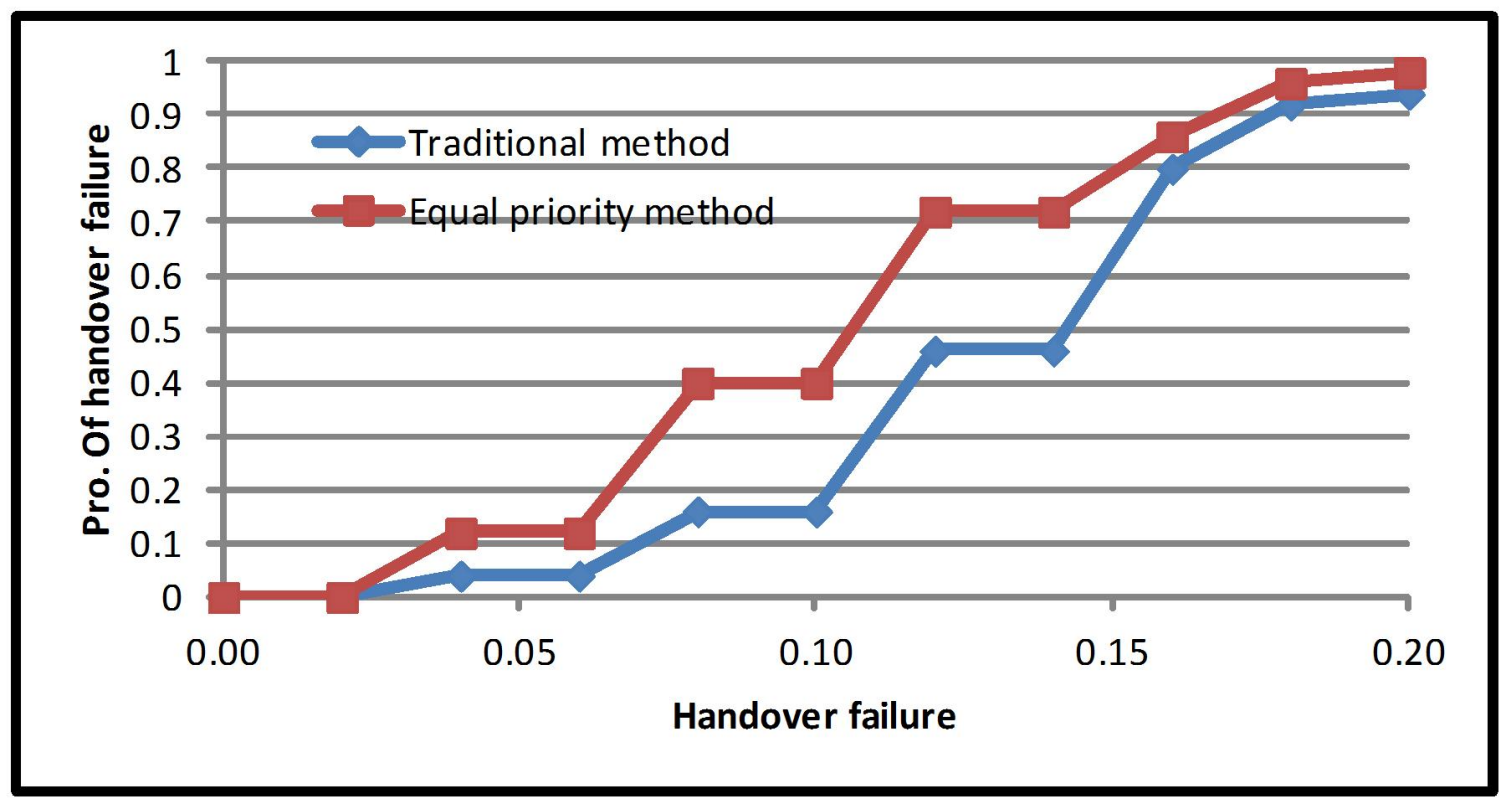
Average handover failure probability of the equal priority method.

Figure 5 presents the average handover failure probability for the equal priority multiple criteria method. Handover failure probability is a fundamental performance metric because it indicates the ability of the network to serve incoming mobile users. However, equal priority multiple criteria improved the average handover failure probability by 24.62%. The average handover failure probability for the traditional method is 0.24, and the average handover failure probability for equal priority multiple criteria is 0.18. Equal priority has an equal proportion for all criteria (mobile criteria and network criteria); meanwhile, the handover failure probability is closely related to the network.

**Figure 6:**
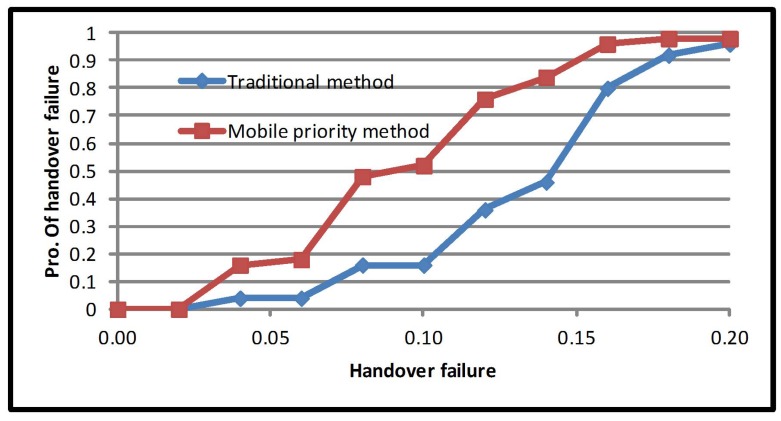
Average handover failure probability of the mobile priority method.

**Figure 7:**
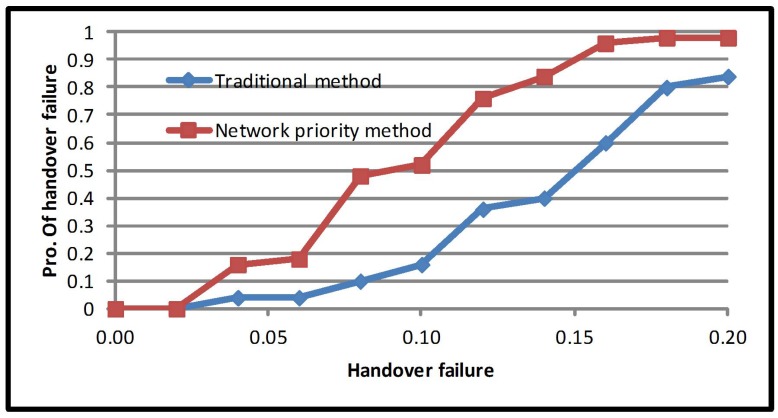
Average handover failure probability of the network priority method.

Apart from that, providing larger network occupancy and mobile priority multiple criteria can improve handover failure probability; the average increased by 33.79%. As demonstrated in Figure 6, the average probability value for the traditional method is 0.27, while for mobile priority multiple criteria, it is 0.18. The network priority multiple criteria significantly improved the average handover failure probability by 43%. Meanwhile, the average handover failure probability is 0.27 for the traditional method and 0.15 for the network priority multiple criteria method. From this, it can be concurred that the average handover failure probability is linked to network occupancy. It should be noted that in the network priority multiple criteria method, the load on the network is higher than RSS, cost function and mobile speed. Hence, prioritizing the network will reduce the average failure probability (Figure 7).

## 6. Analytical Model

**Figure 8:**
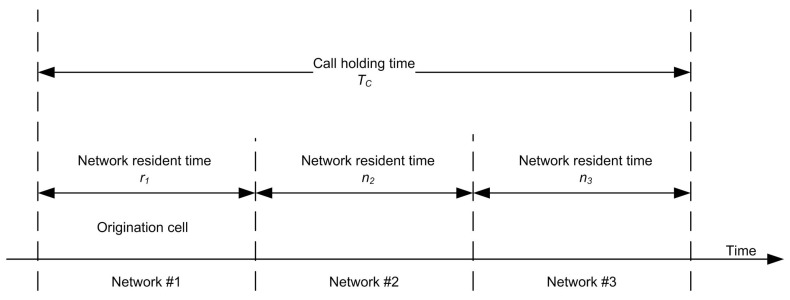
Timing diagram for network residence time and call holding time.

The number of handovers can also be defined as the number of handovers requested during a call connection. Such requests affect the handover arrival traffic and call admission control policy design [29]. This is why the number of handovers is chosen as a parameter to assess algorithms. The definition of call holding time and the call residence time (Figure 8) should be clear in an analytical model.

1. Call holding time ($T_{C}$): the duration from the accepted call instant to the call completed instant.
2. Network residence time in the origination network ($r_{1}$): the mobile user travel duration from the original call point to the edge of the network.
3. Network residence time in the handover network ($t_{n}$, i = 2, 3, 4, …): the mobile user travel duration through a network (edge to edge) reached after (n −1, n = 2, 3, 4,…) handover(s).

This paper will now focus on the configuration of the calculation of the number of handovers.

Let (NH) = the number of a non-blocked calls during a call connection. Then:

1. If the network residence time in the original network r1 is longer than the call holding time ($T_{C}$), NH = 0.
2. If the call is terminated because of the first handover failure or the call makes the first successful handover and is completed successfully in a new network, NH = 1.

Apart from that, a non-blocked call handover probability ($P_{NH}$) is as follows.

(8) $$P_{NH}=\;\left\{\begin{array}{c} \;\;\;P_{NH}\left(T_{C}<r_{1}\right)\;\;\;\;\;\;\;\;\;\;\;\;\;\;\;\;\;\;\;\;\;\;\;\;\;\;\;\;\;\;\;\;\;\;\;\;\;\;\;\;\;\;\;\;\;\;\;\;\;\;\;\;\;\;\;\;\;\;NH=0\\ P_{NH}{\left(r_{1}\;+\;t_{2}+..+t_{NH}\;<\;T_{C}\leq \;r_{1}\right.+\;t_{2}+..+t_{NH+1}\left(1-P_{fh}\right)}^{NH}\;\;\;\;\;\;\;\;\;\;\;\;\;\;\;\;\\ +{P_{NH}\left(T_{C}>\;r_{1}\;+\;t_{2}+..+t_{NH}\right)\left(1-\;P_{fh}\right)}^{NH-1}P_{fh}\;\;\;\;\;\;\;\;\;\;\;\;\;\;\;\;\;\;\;\;\;\;\;\;\;\;\;\;\;\;\;NH\geq 1 \end{array}\right\}\;$$

where $P_{fh}$ is the handover failure probability because of the lack of resources. Then, it can be simplified as follows:

(9) $$P_{NH}=\;\left\{\begin{array}{c} \;\;{1-\;P}_{NH}\left(T_{C}>r_{1}\right)\;\;\;\;\;\;\;\;\;\;\;\;\;\;\;\;\;\;\;\;\;\;\;\;\;\;\;\;\;\;\;\;\;\;\;\;\;\;\;\;NH=0\\ P_{NH}{\left(T_{C}>r_{1}\;+\;t_{2}+..+t_{NH}\right)\left(1-P_{fh}\right)}^{NH-1}-\;\;\;\;\;\;\;\;\;\;\;\;\;\;\;\;\;\;\;\;\;\;\;\;\;\;\;\;\;\;\;\;\;\\ {P_{NH}\left(T_{C}>\;r_{1}\;+\;t_{2}+..+t_{NH+1}\right)\left(1-\;P_{fh}\right)}^{NH}\;\;\;\;\;\;\;\;\;\;\;\;\;\;\;\;\;\;\;NH\geq 1 \end{array}\right\}$$

To find the approximating of network residence times ($r_{1}\;+\;t_{2}+..+t_{NH}$) for mobile nodes, we will use the probability density function (pdf) [29].

Assume that the random variable $R_{NH}$ = ($r_{1}\;+\;t_{2}+..+t_{NH}$). Then:

1. The residence time in the first network $r_{1}$ is the gamma distributed random variable that has the same mean as $R_{r_{1}}$ [30].
2. The residence time in all subsequent networks is the gamma distributed random variable that have the same mean $R_{t_{i}}$ and variance $\sigma_{t_{i}}^{2}$.
  (10) $${pdf}_{r_{1}}\left(t\right)≅\;\frac{\beta_{1}^{\alpha_{1_{t}}\alpha_{1}-\;1}e^{-\beta_{1}^{t}}}{\Gamma (\alpha_{1})}$$
  and:
  (11) $${pdf}_{r_{i}}\left(t\right)≅\;\frac{\beta_{i}^{\alpha_{i_{t}}\alpha_{i}-\;1}e^{-\beta_{i}^{t}}}{\Gamma (\alpha_{i})}$$
  where ${pdf}_{x}\left(t\right)$ is the random variable x and $\Gamma \left(x\right)=\int_{0}^{\infty }t^{x-1}e^{-t}dt$.

Hence, the mean and the variance of the random variable $t_{i}$ may be found from Equation (11), respectively, as:

(12) $$R_{t_{i}}=\;\frac{\alpha_{i}}{\beta_{i}}\;,\;\;\;\;\;\;\;\sigma_{t_{i}}^{2}=\;\frac{\alpha_{i}}{\beta_{i}^{2}}$$

Solving Equation (12) for $\alpha_{i}\;and\;\beta_{i}$, we have:

(13) $$\alpha_{i}\;=R_{t_{i}}\beta_{i},\beta_{i}=\frac{R_{t_{i}}}{\sigma_{t_{i}}^{2}}$$

Similarly, for the residence time in the first network, we have $R_{t_{1}}=\frac{\alpha_{1}}{\beta_{1}}$, and letting $\beta_{1}=\;\beta_{i}$, we have:

(14) $$\alpha_{1}\;=R_{r_{1}}\beta_{1}=\frac{R_{r_{1}}R_{t_{i}}}{\sigma_{t_{i}}^{2}}$$

It can be seen that $R_{NH}$ is the sum of (NH) gamma distributed random variables with the same shape parameter $\beta_{i}$. Hence, $R_{NH}$ is also a gamma distributed random variable with parameters.

(15) $$\alpha_{NH}=\alpha_{1}\left(m-1\right)\alpha_{i},\;and\;\beta =\beta_{1}=\;\beta_{i}$$

Hence, the pdf of $R_{NH}\;$ may be found as:

(16) $${pdf}_{R_{NH}}\left(t\right)=\;\frac{\beta^{\alpha_{NH}}t^{\alpha_{m}-1}e^{-\beta t}}{\Gamma (\alpha_{NH})}$$

From Equation (9), we have:

(17) $$P_{NH}=\;\left\{\begin{array}{c} \;\;1-\;P_{NH}\left(T_{C}>r1\right)\;\;\;\;\;\;\;\;\;\;\;\;\;\;\;\;\;\;NH=0\\ \;{P_{NH}\left(T_{C}>R_{NH}\right)\left(1-\;P_{fh}\right)}^{NH-1}-\;\;\;\;\;\;\;\;\;\;\\ {P_{NH}\left(T_{C}>R_{NH-1}\right)\left(1-\;P_{fh}\right)}^{NH}\;\;\;NH\geq 1 \end{array}\right\}$$

Then:

(18) $$P_{NH}=\;\left\{\begin{array}{c} \;\;1-\int_{0}^{\infty }{pdf}_{R_{1}}\left(t\right)\left(1-D_{T_{C}}\left(t\right)\right)dt,\;\;\;\;\;\;\;\;\;\;\;\;\;\;\;\;NH=0\\ \;{\left(1-P_{fh}\right)}^{NH-1}\int_{0}^{\infty }{pdf}_{R_{NH}}\left(t\right)\left(1-D_{T_{C}}\left(t\right)\right)dt-\;\;\;\;\;\;\;\;\;\\ {\left(1-P_{fh}\right)}^{NH}\int_{0}^{\infty }{pdf}_{R_{NH-1}}\left(t\right)\left(1-D_{T_{C}}\left(t\right)\right)dt\;NH\geq 1 \end{array}\right\}$$

We select the network priority handover decision algorithm, which gives the best results compared to the equal priority and the mobile priority handover decision algorithm for evaluation by use of Equation (11).

**Figure 9:**
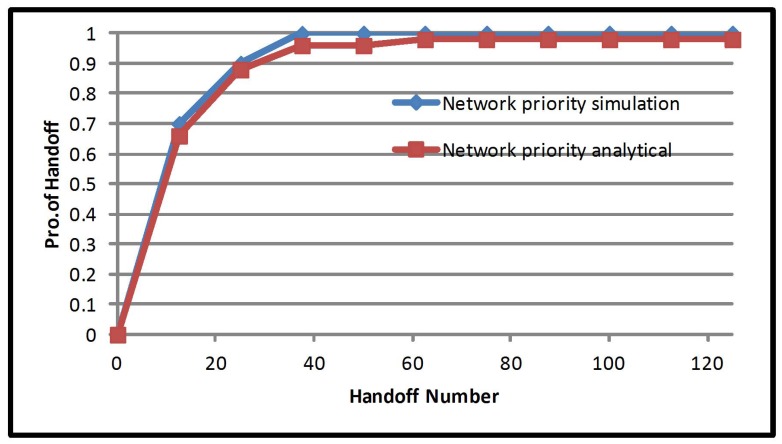
Handoff number probability.

Our method of calculating the probability function of the number of handovers and comparing with simulation results is shown in Figure 9.

The discussion that has been built demonstrates that the analytical results from the introduced technique and computer simulation agree with the different system parameters. The former also leads to the high accuracy of the results.

## 7. Conclusions

To overcome the problem of excessive handovers for mobile devices in the environment of a heterogeneous network, we propose an improved vertical handover decision algorithm based on multiple criteria, which will enable the mobile devices to make the right handover decision. From the simulation results, we can observe that our proposal will enhance the number of handovers’ probability and the handover failure probability in comparison with the traditional network decision algorithm. The network priority handover decision algorithm creates good results compared to the equal priority and the mobile priority handover decision algorithm.

## Figures and Tables

**Table 1:** A comparative summary of the vertical handover decision algorithms.

Categories	Heuristic	Advantages	Disadvantages
RSS-based algorithms	Eshanta et al. [3]	Low complexity	Reduced reliability
	Yan et al. [4]		
	Mohanty et al. [5]		
Context-aware-based algorithms	Zekri et al. [1]	High throughput	Long handover delay
	Maaloul et al. [7]		
	Ahmed et al. [8]		
Cost function-based algorithms	Ong and Khan. [9]	High user satisfaction	Very complex
	Tawil et al. [11]		
	Hasswa et al. [12]		
Fuzzy logic-based algorithms	Xia et al. [13]	High reliability	Very complex
	Nasser et al. [14]		
	Pahlavan et al. [15]		
Multiple criteria-based algorithms	Alsalem et al. [16]	Low handover failure	No support of the fuzzy decision
	Ismail et al. [17]		
	Ismail et al. [18]		

**Table 2:** Priority weights.

Criteria	Equal Priority	Mobile Priority	Network Priority
Cost function	0.25	0.4	0.1
Mobile speed	0.25	0.4	0.1
RSS	0.25	0.1	0.4
Network occupancy	0.25	0.1	0.4
